# The effectiveness of robotic-assisted gait training for paediatric gait disorders: systematic review

**DOI:** 10.1186/s12984-016-0214-x

**Published:** 2017-01-05

**Authors:** Sophie Lefmann, Remo Russo, Susan Hillier

**Affiliations:** 1 Sansom Institute for Health Research, University of South Australia, GPO Box 2471, Adelaide, SA 5001 Australia; 2 Paediatric Rehabilitation Department, Women’s and Children’s Health Network, Women’s and Children’s Hospital Campus, 72 King William Road, North Adelaide, SA 5006 Australia

**Keywords:** Gait, Robot-assisted training, Paediatric, Rehabilitation

## Abstract

**Background:**

Robotic-assisted gait training (RAGT) affords an opportunity to increase walking practice with mechanical assistance from robotic devices, rather than therapists, where the child may not be able to generate a sufficient or correct motion with enough repetitions to promote improvement. However the devices are expensive and clinicians and families need to understand if the approach is worthwhile for their children, and how it may be best delivered.

**Methods:**

The objective of this review was to identify and appraise the existing evidence for the effectiveness of RAGT for paediatric gait disorders, including modes of delivery and potential benefit. Six databases were searched from 1980 to October 2016, using relevant search terms. Any clinical trial that evaluated a clinical aspect of RAGT for children/adolescents with altered gait was selected for inclusion. Data were extracted following the PRISMA approach. Seventeen trials were identified, assessed for level of evidence and risk of bias, and appropriate data extracted for reporting.

**Results:**

Three randomized controlled trials were identified, with the remainder of lower level design. Most individual trials reported some positive benefits for RAGT with children with cerebral palsy (CP), on activity parameters such as standing ability, walking speed and distance. However a meta-analysis of the two eligible RCTs did not confirm this finding (*p* = 0.72). Training schedules were highly variable in duration and frequency and adverse events were either not reported or were minimal. There was a paucity of evidence for diagnoses other than CP.

**Conclusion:**

There is weak and inconsistent evidence regarding the use of RAGT for children with gait disorders. If clinicians (and their clients) choose to use RAGT, they should monitor individual progress closely with appropriate outcome measures including monitoring of adverse events. Further research is required using higher level trial design, increased numbers, in specific populations and with relevant outcome measures to both confirm effectiveness and clarify training schedules.

## Background

Gait disorders in children can result from a number of conditions including cerebral palsy (CP), myelomeningocele, traumatic brain injury or after orthopaedic surgery or musculo-skeletal pathology. Such disorders can have a profound effect on the kinematics and tempero-spatial components of walking, as well as the participation of children in their home, school and other natural environments.

Rehabilitation specialists and physiotherapists are interested in ways to influence sustained improvement in quality, speed, independence and efficiency of gait to enhance participation of children in a variety of different settings. Robotic-assisted gait training (RAGT) has become an increasingly common rehabilitation tool over the last decade to improve the gait pattern of people with neurological impairment [1]. Advances in treadmill and robotic technology have refined ways to support the person’s body weight, while helping the lower limbs and torso to maintain appropriate alignment and patterns when the person initiates and performs gait. The overall intent for people using RAGT is to vary the degree of active participation and degree of body-weight support needed during functional gait, as determined by their therapeutic goals developed with their rehabilitation team [1]. RAGT differs from more traditional body-weight supported treadmill training in that the robotic-controlled exoskeleton or footplates can assist with the specific guidance of hip, knee and ankle movements, instead of the therapist supporting or guiding the person’s body segment positioning [2].

Zwicker and Mayson [3] provided an overview of systematic reviews of all treadmill training studies for children with motor impairments in 2010, finding the majority of reviews have been conducted on treadmill training *per se* (with or without body-weight support). Further, they concluded that the evidence suggests that overall treadmill training with or without support and assistance is effective, but this effectiveness is not as clear for different pathology populations or different ages.

A small number of RAGT devices are on the market or used in clinical research. The Lokomat® and ReoAmbulator ® use a computer-driven exoskeleton to regulate and monitor gait parameters in individuals with a compromised gait pattern [1]. Alternatively, the Gait Trainer GT1® uses a system of moveable footplates to encourage walking, instead of a treadmill-based system [4]. Additional biomechanical parameters of gait can be monitored and recorded, and adjustments to performance made with feedback from the supervising therapist. Increasingly, modes of extrinsic feedback and motivation, such as biofeedback and virtual reality game participation, are being used in conjunction with RAGT to facilitate the participant’s active regulation of their performance [5].

While there is a growing body of evidence on the effectiveness of RAGT in adults (see systematic review for stroke from Swinnen and colleagues [6]), evidence for paediatric populations is not so clear [7]. Aurich and colleagues [7] recognised the need for greater clarity for clinicians to be able to safely apply this technology, and assembled an expert panel to provide recommendations, pending scientific evidence. This systematic review aims to augment these consensus guidelines by providing a relevant summary of the scientific evidence to date, to both inform clinicians and to guide researchers in the quest for more robust evidence. The research question was ‘what is the level of evidence for the effectiveness of the use of RAGT for paediatric gait disorders, in which domains, and using what delivery protocols?’

## Methods

### Operational definition of population and intervention

Experimental studies were examined to determine the current volume and quality of available literature on RAGT in paediatric participants (aged 5–17 years). The search population focused on children with gait disorders of any aetiology (neurological, orthopaedic or developmental). The intervention included any dedicated period of rehabilitation (e.g. more than a single session) using a specified RAGT device (e.g. Lokomat ®, Gait Trainer GT1 ®), where the effect could be distinguished from concomitant therapy. As discussed, RAGT can provide all or part of a naturalistic gait cycle, contrasting body weight support/treadmill training where the participant or the therapist must provide or augment active motion beyond that provided by the device.

### Search strategy

The search strategies were influenced by the PRISMA protocol [8]. A health sciences librarian was consulted for the initial development of the search protocol. Search terms included pediatric OR paediatric; lokomat OR robotic assisted treadmill; child* OR adolescen* OR youth OR young adult* OR teenager*; robotic assisted gait training; gait. Databases searched included CINAHL (1981–2016); EMBASE (1980–2015); Cochrane Database of Systematic Reviews (1993–2016); Physiotherapy Evidence Database (PEDro); MEDLINE (1980–2016); and Scopus (1960–2016). Limiters included age and language. Pearling of the reference lists of identified studies was conducted.

Articles were included if they were:

- experimental studies (e.g. randomized controlled trials (RCT), before/after studies);
- conducted on (majority) paediatric participants ≤17 years; and
- specifically used RAGT as a primary intervention method for > single session.

Articles were excluded if they:

- could not be accessed as an English-language version; or
- were not full-text, original research (e.g. commentaries, conference abstracts).

Any level of experimental research design was considered given the need to determine RAGT’s broad clinical effectiveness. Other research manuscripts (e.g. literature reviews) were considered in the initial stages to facilitate pearling of sources, but were not included in the final summary of evidence.

From the original list of studies generated, one reviewer removed studies that were obviously ineligible based on title. After this first cull, abstracts and/or full texts were then reviewed by two reviewers independently and further exclusions made. Decisions for inclusion/exclusion were based on consensus, and facilitated by the third reviewer as necessary. All authors had articles allocated as first, second or third reviewer equally.

### Data extraction

Two of the three potential reviewers independently extracted data, including a determination of study type and therefore level of evidence (using the NHMRC levels of evidence tables [9]), population characteristics, practice setting, total number of participants, intervention (and comparison), outcomes and results. The reported outcomes were further categorised into the domains stipulated by the International Classification of Functioning, Disability and Health (ICF).

### Risk of bias

Included articles were critically appraised by at least two members of the research team using the risk of bias checklist constructed by the Cochrane Collaboration [10]. This list provides the key aspects of trial design that avoid bias in study conduct including randomising, blinded conduct and adequate reporting. The three authors acted independently as primary or secondary reviewers for each study with the third allocated as consensus arbiter as necessary.

### Data management

All extracted data were reported in table form and as a narrative synthesis. Where appropriate, data were pooled from sufficiently homogenous studies in a meta-analysis [10] to produce summary effects using Revman 5.

## Results

### Organisation of evidence: trial characteristics

**Fig. 1:**
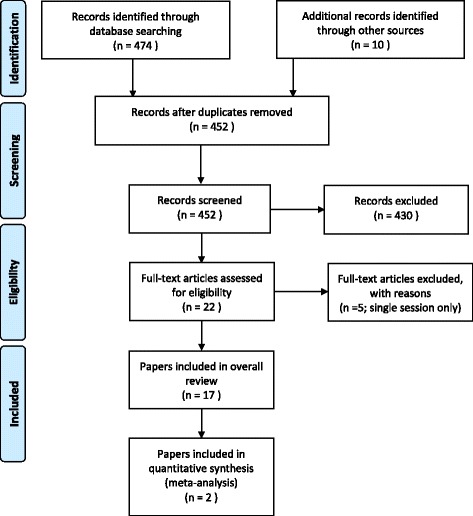
PRISMA flow chart of included studies

Seventeen papers were identified from the search – see Fig. 1 for the flowchart summarising the results of the search. Of the 17 identified, three were RCTs (NHMRC II), one used a non-randomised control group (NHMRC III2), three used an interrupted time-series design (NHMRC III3), and the remaining 10 were pre/post-test design with no control (NHMRC IV). Individual studies and their level of evidence, as described using the NHMRC nomenclature, are summarized in Table 1.

**Table 1:** Summary of studies, in order of level of evidence, including intervention and participant descriptors

Group studies	Level of evidence Research design	Participants	Total n	Ages (y)	Intervention	Control intervention
Druzbicki 2010 [15]	II RCT	CP GMFCS II-IV	18	6–14	RAGT + PT 20 sessions over 4/52 (45 min duration)	Individual PT only
Druzbicki 2013 [18]	II RCT	CP: SD GMFCS II and III	52	10–11	RAGT 20 sessions over 4/52 (45 min duration)	Individual PT only
Smania 2011 [4]	II RCT	CP GMFCS I-IV	18	E = 13 ± 2.8 C = 12 ± 3.1	RAGT 5 sessions (30 min RAGT and 10 min stretching: 40 min duration) over 2/52	5 × 40 min sessions of exercises over 2/52
Nikityuk 2016 [20]	III2 pre/post test with control	CP (average GMFCS level of III	27	E = 9.0 ± 3.67 C = 9.1 ± 4.84	RAGT + FES 15 sessions (30 min duration) over 3/52	RAGT (no FES), duration same as for E
Brutsch 2011 [14]	III3 Interrupted time series (two schedules)	Neurol (10): CP 5, TBI 2, MM 2, SLE 1.	10	12.47 +/− 2.75	RAGT 1 of 2 schedules of feedback (30–40 min duration)	Crossover 2nd of 2 schedules of feedback (30–40 min duration)
Patritti 2010 [5]	III3 Interrupted time series ABA vs ACA	CP: D (GMFCS II *x*2; III *x*2)	4	5.5–8	RAGT + augmented feedback	RAGT (no FB)
Schroeder 2014 [19]	III3 Interrupted time series (ABA)	CP (bilateral spastic) GMFCS I-IV	18	11.5	RAGT (B) 12 sessions (30–60 min duration) over 3/52	Control (A) period before and after usual physio 3/52
Bayon 2016 [17]	IV Pre/post test	CP (bilateral spastic) GMFCS II and III Post-SEMLS (11 & 42 months)	2	12 and 14	RAGT using novel ‘CPWalker’ 10 sessions (60 min duration) over 5/52.	n/a
Borggraefe 2008 [21]	IV Pre/post test Single case	CP GMFCS III	1	6	RAGT 12 sessions over 3/52 (mean 34 min duration)	n/a
Borggraefe 2010 [25]	IV Pre/post test	CP GMFCS I-IV	20	11.0 ± 5.1	RAGT 12 sessions (50 min duration) over 3/52	n/a
Borggraefe 2010 [12]	IV Pre/post test	CP (58) Neurol (20) Orthop (2) Miscel (9)	89	11.4+/−4.5	RAGT 12 sessions over 3/52 (30 min duration)	n/a
Borggraefe 2010 [22] (f/u of [25])	IV Pre/post test	CP (13) Spinal paralysis (1)	14	8.2 ± 5.4	RAGT 12 sessions (50 min duration) over 3/52 plus follow-up sessions 2-3p/m over 6/12	n/a
Koenig 2008 [16]	IV Pre/post test Descriptive case study	CP - nil further data	15	No data	RAGT (different VR scenarios with haptic feedback)	n/a
Meyer-Heim 2007 [13]	IV Pre/post test	CP (19) TBI/GB etc. (7)	26	10.1+/−4	RAGT 3–20 sessions (45 min duration)	n/a
Meyer-Heim 2009 [23]	IV Pre/post test	CP (22) GMFCS II-IV	22	8.6	RAGT 3–5 sessions (45–60 min duration)	n/a
Schroeder 2014 [11]	IV Pre/post test	CP GMFCS I-V	83	10.9 ± 6.1	RAGT 12 sessions (30–60 min duration) over 3/52	n/a
van Hedel 2015 [24]	IV Pre/post test retrospective	CP GMFCS II-IV	67	11.3 ± 4	RAGT At least one session	n/a

*RCT* randomised controlled trial, *CP* cerebral palsy, *GMFCS* gross motor function classification system, *RAGT* robot-assisted gait training, *PT* physiotherapy, *SD* spastic diplegia, *E* experimental group, *C* control group, *FES* functional electric stimulation, *Neurol* neurological, *TBI* traumatic brain injury, *MM* myelomeningocele, *SLE* systemic lupus erythromatosis, *AB, ABA and ACA* indicates types of interrupted time series design, *FB* feedback, *n/a* not applicable, *SEMLs* single event multiple level surgery, *Orthop* orthopaedic, *miscel* miscellaneous, *f/u* follow-up, *VR* virtual reality, *GB* guillain barre

### Participant characteristics

Four hundred and eighty-six participants were recruited across the 17 reports (Table 1). Sample sizes varied from one participant to 89 participants in the included studies. Every study included participants with CP, with the majority rated on the Gross Motor Function Classification System (GMFCS) between level I and IV. Only one study included a participant who had a GMFCS level V [11]. The type and distribution of CP were diverse, with most studies including a variety of presentations including bilateral and unilateral CP of spastic and dyskinetic forms. One study specified its purpose as assessing the RAGT performance of children with CP that had either crouch gait or equinus foot posture (*n* = 4) [5].

Other clinical conditions besides CP were also reported. Borggraefe and colleagues [12] included participants with paraplegia (cause unspecified), Rett Syndrome, traumatic brain injury, Guillain-Barré syndrome, hip dysplasia and patella ‘luxation’ (dislocation). Meyer-Heim and colleagues [13] also recruited participants with Guillain-Barré syndrome, traumatic brain injury and incomplete paraplegia (cause unspecified), as well as haemorrhagic encephalitis and stroke. Brutsch and colleagues [14] reported participants having myelomeningocele, systemic lupus erythematosus and traumatic brain injury as well as CP.

All studies documented an age range, and the mean age across the studies (including control groups) was 9 years 9 months. The studies by Druzbicki and colleagues [15] and Koenig and colleagues [16] were not included in this calculation as no mean age was reported and could not be independently determined.

### RAGT intervention schedules

The heterogeneous aims and methods across trials meant no consistent RAGT schedule was described – the schedules (dosages) are summarized in Table 1 for both the experimental and control groups. Fifteen studies used the Lokomat ® brand of RAGT, one study used the Gait Trainer GT I ® [4] and one used a novel mobile RAGT device called the CPWalker [17]. Most studies described an intervention schedule of between 2 and 5 training sessions per week, over a 2–6 week testing period. Principal aims of these studies were to investigate the improvement of gross motor performance, or kinematic/tempero-spatial performance in the ICF body structure and function domain, from the use of RAGT. Five studies were found and excluded as they described participants’ involvement in a single RAGT session.

Participants’ total time spent on RAGT devices was inconsistent. Testing schedules reflected a typical therapy session duration, between 25 and 60 min and may have included other warm-ups and stretching components. No clear evidence-based guidelines regarding the development of training schedules were articulated. For example, there was little discussion around the use of exercise parameters (e.g. VO_2_ max, heart rate) to determine a personalised training schedule for participants, with most studies appearing to use the RAGT device ‘as tolerated’ by the participant.

A control intervention was only employed in seven of the studies and included variations such as an alternate form of feedback, [5, 14] individual physiotherapy only [15, 18, 19], alternative exercises [4] or combined with functional electrical stimulation [20].

### Risk of bias

Risks of bias assessments for the studies are detailed in Table 2. Scores were generally indicative of extremely high risk of bias, with the major issues being lack of randomization (both generation and concealment, without a control group), as well as conduct issues of poor blinding of assessors, and poor reporting of data (Table 2). There were general sampling issues – mostly samples of convenience from existing clinics and small numbers with no sample size indications for the power of the study. Clearly no study achieved blinding of participants or personnel administering the intervention.

**Table 2:** Risk of bias rating of studies

Study	Level	Random allocation	Allocation concealment	Blinding participants	Blinding of assessment	Incomplete o/come data	Selective reporting	Other
Druzbicki 2010 [15]	II	**+**	**-**	**-**	**+**	**-**	**+**	4 ‘resigned’
Druzbicki 2013 [18]	II	**+**	**-**	**-**	**+**	**-**	**+**	17 ‘resigned’
Smania 2011 [4]	II	**+**	**+**	**-**	**+**	**+**	**+**	
Nikityuk 2016 [20]	III2	**-**	**-**	**-**	**-**	**-**	**+**	
Brutsch 2011 [14]	III3	**-**	**-**	**-**	**-**	**+**	**+**	Time series
Patritti 2010 [5]	III3	**-**	**-**	**-**	**-**	**+**	**+**	Case studies
Schroeder 2014 [19]	III3	**-**	**-**	**-**	**-**	**+**	**+**	Sample issues
Bayon 2016 [17]	IV	**-**	**-**	**-**	**-**	**-**	**+**	
Borggraefe 2008 [21]	IV	**-**	**-**	**-**	**-**	**+**	**+**	Case study
Borggraefe 2010 [25]	IV	**-**	**-**	**-**	**-**	**+**	**+**	Sample issues
Borggraefe 2010 [12]	IV	**-**	**-**	**-**	**-**	**+**	**+**	
Borggraefe 2010 [22]	IV	**-**	**-**	**-**	**-**	**-**	**+**	Lost to f/u
Koenig 2008 [16]	IV	**-**	**-**	**-**	**-**	**+**	**+**	Single case
Meyer-Heim 2007 [13]	IV	**-**	**-**	**-**	**-**	?	**+**	Sample issues
Meyer-Heim 2009 [23]	IV	**-**	**-**	**-**	**-**	**-**	**+**	Sample issues
Schroeder 2014 [11]	IV	**-**	**-**	**-**	**-**	**-**	**-**	Retrospective
Van Hedel 2015 [24]	IV	**-**	**-**	**-**	**-**	**-**	**-**	Retrospective

+ = low; ? = unclear; − = high risk of bias; f/u follow-up

### Outcome measures

A variety of outcome measures were reported in the studies, some standardised and some self-developed. The outcome measures used, their ICF domains and a summary of findings are represented in Table 3. The most common measures used were related to mobility, such as the 6 min walk test (6MinWT) and 10 m walk test (10MWT) [4, 5, 13, 19, 21–24], and the Gross Motor Function Measure-66 (GMFM-66: items D ‘standing’; E ‘walking’) [5, 11, 13, 19, 21–25]. Other mobility-related outcomes included functional walking capability, and independence or level of assistance for gait (functional ambulation classification (FAC)) [13, 23, 24].

**Table 3:** Summary of studies: outcomes, measures and results

Group studies	Outcome of interest	Measure	Component of health		
			Body structure/function	Activity and participation	Contextual factors
Druzbicki 2010 [15]	Balance	Parameters of balance using balance platform	Exp 5/14 parameters improved (*p* < 0.05). Control – 0/14 (NSD)		
Druzbicki 2013 [18]	Gait	3D gait		NSD	
Smania 2011 [4]	Gait speed Distance Gait Function	10MWT 6MinWT 3D gait WeeFIM		*p* = 0.008 *p* = 0.008 NSD NSD	
Nikityuk 2016 [20]	Balance	Stabilometry Plantography	Improvements in RAGT plus FES superior to RAGT alone		
Brutsch 2011 [14]	Joint motion Engagement in therapy	Biofeedback values Self-designed questionnaire: enjoyment of VR	*p* < 0.001 Enjoyed DVD most, but led to reduced engagement		
Patritti 2010 [5]	Distance Gait speed Standing Walking Gait	6MinWT 10MWT GMFM 66: D GMFM 66: E 3D gait		NSD Better with augmented f/b NSD	
Schroeder 2014 [19]	Standing Walking Gait speed Distance Self-goals	GMFM 66: D GMFM 66: E 10MWT 6MinWT COPM		*P* < 0.01^a^ *P* < 0.01^a^ NSD NSD *P* < 0.05	
Bayon 2016 [17]	Gait Range of movement	3D gait Trunk rotation &hip flex/ext	‘specific developments’	‘improved’	
Borggraefe 2008 [21]	Distance Gait speed Standing Walking	6MinWT 10MWT GMFM 66: D GMFM 66: E		55 m to 115 m 0.25 to 0.6 m/s 12.8 to 25.6% 18.1–26.4%	
Borggraefe 2010 [25]	Standing Walking	GMFM 66: D GMFM 66: E		p−0.001 *p* < 0.001	
Borggraefe 2010 [12]	Adverse events	Number Significance	47 AE in 38 pts Mild−moderate		
Borggraefe 2010 [22] (6/12 follow-up)	Standing Walking Gait speed Distance	GMFM 66: D GMFM 66: E 10MWT 6MinWT		*P* < 0.05 *P* < 0.05 *P* < 0.05 *P* < 0.05	
Koenig 2008 [16]	Realism of feedback	Questionnaire	‘Satisfactory impression’		
Meyer-Heim 2007 [13]	Mobility Gait speed Standing Walking Gait assist Distance	Walk time, walk distance 10MWT GMFM 66: D GMFM 66: E FAC 6MinWT		NSD *p* < 0.001 (inpt) *p* = 0.01 (outpt) *p* < 0.05 (inpt) *p* < 0.01 (in/outpt) NSD	*p* < 0.05
Meyer-Heim 2009 [23]	Gait speed Standing Walking Gait assist Distance	10MWT GMFM 66: D GMFM 66: E FAC 6MinWT		*P* < 0.01 *P* = 0.05 NSD NSD	NSD
Schroeder 2014 [11]	Standing Walking	GMFM 66: D GMFM 66: E		*P* < 0.05^a^ *P* < 0.05^a^	
Van Hedel 2015 [24]	Function Gait speed Standing Walking Gait assist Distance	WeeFIM 10MWT GMFM 66: D GMFM 66: E FAC 6MinWT		*P* = 0.003 *P* = 0.018 NSD NSD NSD	NSD

*Exp* experimental group, *NSD* no significant difference between experimental and control group outcomes, *3D gait* 3 dimensional gait analysis, *10MWT* 10 m walk test, *6MinWT* 6 minute walk test, *Wee FIM* functional independence measure for children, *FES* functional electric stimulation, *VR* virtual reality, *DVD* digital video disc, *GMFM 66: D* gross motor function measure 66 part D, *GMFM 66: E* gross motor function measure 66 part E, *f/b* feedback, *COPM* Canadian occupational performance measure, *flex/ext* flexion/extension, *AE* adverse event, *FAC* functional ambulation classification, *inpt* inpatient, *outpt* outpatient, ^a^ = minimum clinically important difference

Other outcomes included motivation to participate, balance and gait parameters (from posturography and 3D analyses), range of motion, motivation to participate, general levels of function (WeeFIM), as well as documenting adverse effects from the use of RAGT.

### Main results

**Fig. 2:**
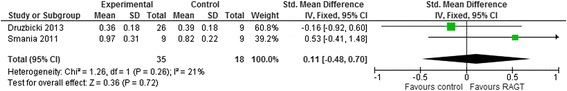
Forest plot of RAGT versus usual physiotherapy on walking speed

Most outcomes reported in individual studies were in the activity domain; as mentioned these were predominantly for the activity of walking. Eight studies assessed children’s preferred walking speed following RAGT. Considering firstly the meta-analysis, we were able to pool data from two of the three RCTs for the outcome measure of gait speed [4, 18]. We found no significant difference between RAGT and the comparison (exercise or physiotherapy alone) (standardised mean difference of 0.11, 95% confidence intervals −0.48–0.70; *p* = 0.720. See Fig. 2). For the remaining individual studies all (except Schroeder [19]) showed that participants’ use of RAGT led to improvements in 10mWT performance with the studies by Smania and colleagues [4], Patritti and colleagues [5], Borggraefe and colleagues [21, 22] indicating that the changes were statistically significant at 1, 3, 4 and 6 months follow-up respectively.

Functional gross motor performance was assessed by nine studies using the GMFM-66, items D (standing) and/or E (walking). All studies except van Hedel [24] showed statistically significant improvements in item D (standing) of the GMFM. Six studies showed statistically significant improvements in item E following RAGT [11, 13, 19, 21, 22, 25]. Three of the eight studies evaluating children’s walking endurance (6MinWT) noted statistically significant improvements [4, 21, 22].

The only participation measure used was the Canadian Occupational Performance Measure (COPM) [19] which reported significant differences post intervention.

For the domain of body structure and function, two studies evaluated the changes in children’s balance performance following combined physiotherapy or FES and RAGT, measured using a stabilometric platform [15, 20]. For the Druzbicki study [15], statistically significant improvements were noted in five of 14 balance domains assessed in the experimental group (*n* = 9), compared with no significant difference in any balance domains in the control group who received physiotherapy intervention only (*n* = 5). The other study [20] compared RAGT with RAGT plus FES and found a greater percent improvement in balance measures with the combination. Three studies reported the enhanced effect of facilitating active engagement among children using RAGT through ‘augmented feedback’; notably either through the use of virtual reality games (e.g. soccer) or therapist encouragement to help children change their level of response to different training demands during RAGT [5, 14, 16].

No studies reported on any aspects of cost or cost-benefit in relation to alternate forms of therapy.

### Adverse events and effects

Table 4 summarises adverse events as reported by one trial. Borggraefe and colleagues [12] conducted the sole study with the primary focus on documenting adverse events among their cohort. They report 47 adverse events among 38 of their 89 participants, with examples including muscle and joint pain, skin erythema and skin lesions, often at the harness site. Five incidents were reported as ‘mild-to-moderate’ [12], and overall very few participants were excluded from RAGT due to an adverse event. No other studies reported this information.

**Table 4:** Reported adverse events (1 study reported; all others not stated)

Study	Level of evidence	Total n	Method of ascertaining adverse event	Description of AEs reported (n of cases)
Group design				
Borggraefe 2010 [12]	IV	89	Active Self-report – confirmed by examination. Number, type and significance.	*n* = 47 (38 pts) muscle (16) and joint pain (14), tendinopathy (1), skin erythema (12) skin lesions (4). *n* = 5 mild-to-moderate

*n* number, *AE* adverse event, *pts* participants

## Discussion

### Clinical applications and areas for future research

Overall there is weak and inconsistent evidence for the effectiveness of RAGT for children with gait disorders. The majority of the evidence is for children with CP and for the benefit of improving gait attributes such as speed, standing ability and in some instances walking distance. However the studies are generally of low level design, with a small sample size and poor conduct ratings. Therefore a clinical recommendation to adopt RAGT in children and adolescents with gait impairment cannot be made until more consistent findings are reported in larger RCTs.

The disparate aims and training schedules used in the existing literature further complicate the development of appropriate recommendations for the use of RAGT with children. At this stage it is unclear from the scientific evidence what intensity, duration or timing of RAGT is best. Borggraefe and colleagues [22] do provide evidence that a relatively intense program over 12 weeks does seem to produce sustained benefit after 6 months however there was no control group so the effects of time alone are not known. Interestingly, children who participate in RAGT do particularly well when virtual games (e.g. soccer) or therapist encouragement are used to enhance their active participation [5, 11, 14]. While not inferring that the use of virtual games is essential for successful RAGT, it does highlight that active participation may be impeded when children’s focus is on more passive media, such as DVDs that require little engagement [14]. Rehabilitation strategies have been shown to produce measureable neuroplastic changes in the cortex of children with hemiplegic CP associated with improvement in upper limb motor skills [26]. These improvements appeared to be more consistent with active, as opposed to passive interventions. Clinicians should consider the type of feedback and motivational tools required to maintain children’s attention on their walking and body placement during RAGT for maximum exercise benefits and active motor learning.

The available evidence has not yet comprehensively highlighted the effectiveness of RAGT at different life stages. For example, the effect of training on children in their early stages of acquisition of walking compared with teenagers experiencing pubertal growth who already have established gait patterns. Van Hedel and colleagues [24] provide some data that suggest that children with CP who are more severely affected may benefit more than those who are less affected – particularly in the areas of walking-related outcomes. As mentioned, the majority of the studies were either solely children with CP or mixed groups. Further research needs to study well-defined groups across a range of diagnoses. Other determinants of success have been suggested as age and gross motor abilities [11, 19] at baseline. While the evidence suggests RAGT may have significant clinical utility and enjoyment among children, training protocols and baseline standards of its use would be useful information for clinicians so that guidelines are evidence-based and not ad hoc.

It appears that the effectiveness of RAGT for children’s gait can be measured with commonly used, commonly available, activity-based outcome measures such as the GMFM or the 10MWT and that in some cases these can be confirmed as clinically meaningful changes. Based on these findings, it is recommended that clinicians use these available tools to track their clients’ changes, especially in children with CP. Schroeder and colleagues [11] provide some early evidence for benefits in the participation domain (using the COPM) however it was a small sample and a time series control, therefore this needs replication in an RCT. Children with CP who participated in an aerobic exercise fitness program showed improvements in physiological measures of aerobic activity [27], but this does not appear to generalise into daily functional activity [28] or in their level of participation or quality of life [29]. Children’s fitness levels and strengthening would seem to be important targets for an RAGT experience, but how this will impact on their activity and participation levels should be a focus of future research. There is a general paucity of research assessing RAGT outcomes on children’s involvement in the “personal factors”, “participation” and “environmental” domains of the ICF. The translation of gait outcomes into children’s meaningful life roles and environments following RAGT is critical information for clinicians to know, as this can shape the goals developed with families and children.

Finally it is of concern that only one study monitored adverse events. Whilst the majority of those events reported were minor (skin redness etc.) the rate was high at 42%. Further research is needed to ascertain rates and to investigate preventive measures for the 5% reported to have adverse events sufficient to cause them to cease the RAGT session.

## Conclusion

There is weak and inconsistent evidence that RAGT may hold benefits for children with gait disorders, particularly those with CP, who wish to improve their walking speed or standing ability. In the absence of clear evidence for training prescription and delivery, clinicians using RAGT should apply clinical judgement and monitor individual client progress closely with appropriate mobility measures. Further research is required using higher level trial design, and increased numbers to confirm effectiveness, across a broad range of outcomes including adverse events, and to clarify training schedules and evidence in different populations, domains or ages.

## Abbreviations

10MWT: 10 metre walk test
6MinWT: 6 minute walk test
COPM: Canadian occupational performance measure
CP: Cerebral palsy
FAC: Functional ambulation classification
GMFCS: Gross motor function classification system
GMFM-66: Gross motor function measure-66
ICF: International classification of functioning, disability and health
RAGT: Robotic-assisted gait training
RCT: Randomised controlled trial
